# Health-related quality of life in vibrant soundbridge patients: generic and specific measures, short-term and long-term outcomes

**DOI:** 10.1007/s00405-024-08889-2

**Published:** 2024-08-23

**Authors:** Franz Muigg, Philipp Zelger, Sonja Rossi, Heike Kühn, Joachim Schmutzhard, Simone Graf, Viktor Weichbold

**Affiliations:** 1 Speech & Voice Disorders, University Hospital for Hearing, Medical University of Innsbruck, Innsbruck, Austria; 2 ICONE – Innsbruck Cognitive Neuroscience, Medical University of Innsbruck, Innsbruck, Austria; 3 Comprehensive Hearing Center Würzburg, University-ENT-Hospital, Würzburg, Germany; 4 Department of Otorhinolaryngology-Head and Neck Surgery, Medical University of Innsbruck, Innsbruck, 6020 Austria

**Keywords:** Audiology, Active middle ear Implant, Quality of life

## Abstract

**Objective:**

The goal of the study was to determine the short- and long-term outcome of health-related quality of life (HRQoL) in adults implanted with a Vibrant Soundbridge (VSB).

**Methods:**

Twenty-one adults (8 females, 13 males; mean age at implantation: 57 ±10 years) who received a unilateral VSB for combined or conductive hearing loss, were administered two questionnaires: the Nijmegen Cochlear Implant Questionnaire (NCIQ) as a measure of hearing-specific HRQoL, and the Health Utility Index 3 (HUI 3) as a measure of generic HRQoL. The questionnaires were administered before implantation and three, six, 12 and 24 months after processor activation.

**Results:**

The NCIQ total score raised significantly from 62 points before implantation to 76 points at three months after processor activation (*p* < 0.005). Thereafter, no significant increases occurred anymore. The HUI 3 multi-attribute score (MAUS) increased from 0.59 before implantation to 0.70 at three months and at six months after processor activation and then declined slightly to 0.68 at 24 months after processor activation. Similar values were observed with the HUI 3 single-attribute score (SAUS) of *Hearing*. The increases of the HUI 3 scores were not statistically significant, but all pre-post-implantation differences were clinically relevant.

**Discussion:**

VSB recipients experienced a quick improvement of their HRQoL. After just three months of device use, a significant improvement of hearing-specific HRQoL and a clinically relevant improvement of generic HRQoL were seen. After three months, no essential changes of HRQoL occurred in our sample, suggesting that the achieved level of HRQoL may remain stable in the long term.

## Introduction

The Vibrant Soundbridge^®^ (VSB) is an active middle-ear implant used mainly for the treatment of conductive or mixed hearing loss (HL), in special cases also for the treatment of sensorineural HL [1–4]. Safety and efficacy of the VSB have been addressed by numerous studies, which were systematically reviewed by Bruchhage et al. [5], by Ernst et al. [6] and, most recently, by Alshalan & Alzhrani [7].

Bruchhage et al. [5] reviewed 22 studies reporting outcomes of the VSB in sensorineural HL. Ernst et al. [6] reviewed 36 publications about VSB in *conductive and mixed* HL. Alshalan & Alzhrani [7] reviewed 27 studies about VSB outcomes in *congenital aural atresia*. All of the reviewing authors concluded that, according to study findings, the VSB is a reliable device, which significantly improves speech perception, delivers good sound quality, offers high wearing comfort and is safe with regard to surgical complications.

As for health-related quality of life (HRQoL) of VSB patients, however, the results of the reviews were less informative. Although some of the reviewed studies assessed subjective outcomes, none of them applied measures of HRQoL. This is true also for studies published after the above-mentioned reviews and therefore not included in them [8–11]. Of the various measurement instruments used for this purpose (e.g. the Abbreviated Profile of Hearing Aid Benefit [APHAB], the International Outcome Inventory [IOI], the Speech Spatial and Qualities of Hearing Scale [SSQ12], the Glasgow Benefit Inventory [GBI]), only the GBI can be considered, in some sense, as a measure of HRQoL. Yet the GBI is designed to compare the outcomes of different interventions and is therefore usually administered *after* an intervention has occurred. Hence, the GBI may not provide reliable data for pre-post comparison of HRQoL, especially not in a long-term perspective.

To our knowledge, only two studies assessed pre-post changes of HRQoL in VSB patients using measures of HRQoL: Edfelt et al. [12] and Lasaletta et al. [13]. Both studies used the Health Utility Index (HUI 3) which is a questionnaire for assessing generic HRQoL; Lasaletta et al. used additional instruments for assessing hearing-specific HRQoL.

Edfeld et al. [12] administered the HUI 3 to 25 VSB recipients (15 with sensorineural HL and 10 with mixed or conductive HL) at two intervals: before and six months after processor activation. They observed a pre-post increase of the HUI total score (also called multi-attribute utility score, MAUS) from 0.57 to 0.66. Although this difference was not statistically significant, it was clinically relevant. Similarly, the score of the single attribute *Hearing* (called single-attribute utility score, SAUS) increased from 0.59 before implantation to 0.73 thereafter, which was both statistically significant and clinically relevant.

Lasaletta et al. [13] reported HUI 3 scores from a large sample of hearing implant recipients, among them 13 VSB recipients with different types of HL. The HUI 3 was administered at three time points: before processor activation and six months and 12 months thereafter. During this period, the HUI 3 total score (MAUS) of VSB recipients increased from 0.60 before processor activation to 0.69 at six months and to 0.75 at 12 months after processor activation. The differences, however, were not statistically significant.

Additionally to the HUI 3, Lasaletta et al. [13] administered the SSQ12 and the Nijmegen Cochlear Implant Questionnaire (NCIQ) to their sample, both – as they state – as measures of hearing-specific QoL. We do not agree that the SSQ12 is a measure of HRQoL (it is rather a measure of listening performance), but the NCIQ definitely is. However, Lasaletta et al. did not administer the NCIQ to VSB patients, because – as they argue – the NCIQ was developed specifically for cochlear implant patients. For this reason, their article does not report NCIQ results from VSB patients.

Our literature search shows that data on HRQoL in VSB patients are still scarce. Long-term data (> 12 months) and short-term data (< 6 months) are completely lacking. Short-term data can be particularly interesting: since the VSB transmits sound to the inner ear in a natural way, it can be assumed that the patients will easily adapt to the device and therefore quickly experience an improvement of their health-related quality of life. Against this background, the aim of our study was to collect additional data on HRQoL in VBS patients, with an observation period that included both early and late measurement points.

In assessing HRQoL, two different concepts of HRQoL were applied in our study: hearing-specific HRQoL and generic HRQoL. To assess the latter, a generic utility-based measure was used (the HUI 3). Such measures do not only provide a concise overview of a person’s HRQoL but have also the potential to influence resource allocation decisions in health care policy making when considered in an economic evaluation [14]. – For assessment of hearing-specific HRQoL, the NCIQ was used. Although the NCIQ was developed specifically for patients with cochlear implants (as Lasaletta et al. [13] point out), this does not exclude that it can also be administered to VSB recipients, since all of its 60 items apply to these patients as well.

## Method

### Study design

The study was conducted as an analysis of questionnaire data obtained routinely within the hearing implant program implemented at the University Clinic for Hearing Speech and Voice Disorders in Innsbruck, Austria, and at the ENT University Clinic in Würzburg, Germany. Approval from the local institutional Ethics Committees for conducting the study was received (ethics approval number AN2016-0212 367/4.9). Within the hearing implant program, two different HRQoL questionnaires (see below for description) were administered to the patients at the following five points in time: before implantation and three, six, 12 and 24 months after audio processor activation.

### Sample

The study sample consisted of 21 adults (13 males, 8 females) who received a unilateral VSB for conductive or mixed hearing loss in one of the above named clinics. The patients were implanted between September 2015 and December 2016 (study onset) and were followed over a period of two years for data collection. Only VSB patients were included in the study who were aged 18 years or older, had adequate speech-language and cognitive skills, and had provided informed consent to study participation.

The mean age of the sample at the time of implantation was 57.0 (±10.2) years. The patients’ ages ranged from 38.0 to 73.4 years. Five of them had a conductive HL and 16 had a mixed HL. Causes for their HL were: (a) multiple middle ear operations after cholesteatoma, radical cavity, tympanoplasty (*n* = 10); (b) otosclerosis (*n* = 4); (c) no hearing aid possible due to chronic infections or eczema in the outer ear canal (*n* = 5); (d) aural atresia (*n* = 2). Air conduction thresholds were, on average (pure-tone-average of hearing threshold levels at four frequencies: 500, 1000, 2000 and 4000 Hz), 67.0 (±19) dB HL in the ear that became implanted, and 34.1 (±17) dB HL in the contralateral ear. Averaged bone conduction thresholds were 33.3 (±15) dB HL in the implanted ear, and 25.7 (±12) dB HL in the contralateral ear. None of the patients included in the sample discontinued device use during the two-year observation period of this study.

### Questionnaires

Two questionnaires were used to assess HRQoL in the sample: the Nijmegen Cochlear Implant Questionnaire (NCIQ) and the Health Utility Index 3 (HUI 3).

The NCIQ [15] has been developed for assessment of *hearing-specific* HRQoL in cochlear implant patients. It nevertheless applies to patients with other hearing implants as well as all of its items address behaviours related to hearing performance. The 60 items of the NCIQ cover six domains of hearing-specific HRQoL: Basic Sound Perception (BSP), Advanced Sound Perception (ASP), Speech Production (SP), Self-Esteem (SE), Social interaction (SI), and Activity (Act). Responses to items are given on a five-step scale with the values 0 / 25 / 50 / 75 / 100. Internal consistency (Crohn’s alpha) of the six domains has been reported to range from 0.73 < α < 0.89; retest reliability has been reported to range from 0.64 < *r* < 0.85 [15].

The HUI 3 [16, 17] is a questionnaire for measuring generic and attribute-specific HRQoL and producing utility scores. It includes eight attributes (domains) which are considered to cover the full range of abilities/disabilities relevant to the human health status: Vision, Hearing, Speech, Ambulation, Dexterity, Emotion, Cognition, Self-care and Pain. The HUI 3 scoring system provides utility scores on a generic scale between values 1.00 (“perfect health”) and 0.00 (“dead”). Utility scores can be computed for each of the eight attributes (single-attribute utility score, SAUS) and for the general health-state (multi-attribute utility score, MAUS). Further information about the HUI 3 is summarized and reviewed in Horsman et al. [17].

### Statistical analysis

NCIQ total scores obtained at the five points in time were tested for change over time with a repeated measures ANOVA (Analysis of Variance). Sphericity assumption was tested with the Mauchly test and, if violated, Greenhouser-Geisser correction was applied to degrees of freedom. Alpha-error level was set at 5%. Post-hoc comparisons were performed with the paired-sample t-test at a Bonferroni-adjusted alpha-error level α’ = (0.05 / 10 =) 0.005.

The same tests were performed with the HUI 3 single-attribute utility score (SAUS) of the attribute *Hearing*, and with the HUI 3 multi-attribute utility score (MAUS). Differences between HUI scores were also interpreted in terms of clinical relevance. It has been proposed to consider a difference of 0.03 or more between mean scores as clinically important [14, 17, 18].

### Treatment of missing data

Some of our VSB patients did not attend all five measurement sessions. The reason was that the measurement sessions were combined with the fitting of the audio processor, but the patients did not show up for processor fitting because they were satisfied with the way their device worked. This reason for non-attendance suggests that the patients’ HR-QoL had at least not deteriorated. Therefore, the missing questionnaire data were substituted with the “Last Observation Carried Forward (LOCF)” method. With this method, a missing value is replaced by the value measured at the immediately preceding measurement session.

## Results

### Hearing-specific HRQoL (NCIQ)

Table 1 provides the mean scores of the NCIQ obtained at the five measurement time-points, shown as total mean score (last line) and as domain-specific mean scores (lines above). The NCIQ total mean score (last line) increased markedly between measurement-points *pre-operative* and *3 months after processor activation* and underwent only minor fluctuations at subsequent time-points. Repeated measures ANOVA showed significance for the change over time: F = 12.47, df = 1.99, *p* < 0.001. Pairwise comparisons of the scores from the five measurement-points revealed that the pre-operative total score differed significantly from all others (with all p‘s < 0.005), but no significant differences existed between the other scores. This finding indicates that hearing-specific HRQoL improved within three months of device use to a significant extent, and afterward remained (more or less) stable.

**Table 1:** Domain-specific mean scores and total mean score of the NCIQ at the five measurement time-points. In brackets: standard deviations

NCIQ-Domains	pre	3 months	6 months	12 months	24 months	3-months-change	2-year-change
Basic Sound Perception	64.6 (17.2)	76.7 (16.6)	76.6 (18.8)	78.1 (17.7)	77.2 (15.9)	*+ 12.1*	*+ 12.6*
Advanced Sound Perception	69.3 (17.0)	81.6 (15.6)	80.6 (15.8)	83.8 (13.9)	81.3 (14.2)	*+ 12.3*	*+ 12.0*
Speech Production	74.9 (14.3)	83.3 (10.9)	83.3 (11.7)	85.9 (9.7)	86.5 (10.4)	*+ 8.4*	*+ 11.6*
Self-Esteem	51.1 (14.8)	68.2 (15.7)	64.5 (18.8)	67.9 (16.7)	70.7 (16.1)	*+ 17.1*	*+ 19.6*
Activity	57.1 (18.3)	75.6 (21.1)	72.3 (18.0)	76.9 (18.5)	77.3 (17.8)	*+ 18.5*	*+ 20.2*
Social Interaction	57.0 (14.2)	70.9 (18.9)	70.3 (19.1)	74.2 (17.4)	78.8 (16.5)	*+ 13.9*	*+ 21.8*
Total Mean Score	62.3 (13.9)	76.1 (14.6)	74.6 (14.7)	77.8 (13.6)	78.6 (13.0)	*+ 13.8*	*+ 16.3*

A similar picture is seen when looking at the domain-specific mean scores of the NCIQ. On each of the six domains, the most important gain is made within the first three months of VSB use. Thereafter, the scores are only slightly fluctuating – with exception of those of the subdomains *Social Interaction* (SI) and *Speech Production* (SP). In these two domains, the scores continue to increase up to 24 months. Statistical testing reveals that (a) the increases between *before implantation* and *three months after implantation* are significant in all six domains (with all p’s < 0.001) and that (b) in the domain *Social Interaction* the increases between three months and 24 months, and between six months and 24 months, are also significant (*p* < 0.005). All other differences are not significant.

### Generic HRQoL (HUI 3)

The level of generic HRQoL is indicated by the multi-attribute utility score (MAUS) of the HUI 3. In addition, the HUI 3 allows for computing a single-attribute utility score (SAUS) for each of the eight health attributes *Vision*,* Hearing*,* Speech*,* Ambulation*,* Dexterity*,* Emotion*,* Cognition*, and *Pain*. While the MAUS is an indicator of the general health status, the SAUS is an indicator of the functional state of a single health attribute. The MAUS and the SAUS of the eight attributes found in our sample are shown in Table 2.

**Table 2:** Single-attribute utility mean scores (SAUS) of the eight attributes and multi-attribute utility mean score (MAUS) of the HUI 3. In brackets: standard deviations

HUI-Scores	pre	3 months	6 months	12 months	24 months	3-months-change	2-years-change
SAUS Vision	0.94 (0.12)	0.96 (0.06)	0.98 (0.03)	0.95 (0.09)	0.95 (0.06)	*+ 0.02*	*+ 0.01*
SAUS Hearing	0.59 (0.32)	0.70 (0.10)	0.68 (0.14)	0.72 (0.13)	0.71 (0.13)	*+ 0.11*	*+ 0.12*
SAUS Speech	0.96 (0.09)	1.00 (0.01)	1.00 (0.01)	1.00 (0.01)	0.98 (0.08)	*0.00*	*+ 0.02*
SAUS Ambulation	0.96 (0.07)	0.98 (0.05)	1.00 (0.01)	0.95 (0.08)	0.98 (0.06)	*+ 0.02*	*+ 0.02*
SAUS Dexterity	0.99 (0.04)	0.97 (0.05)	0.99 (0.04)	0.95 (0.13)	0.98 (0.05)	*+ 0.02*	*-0.01*
SAUS Emotion	0.94 (0.07)	0.96 (0.07)	0.94 (0.07)	0.92 (0.15)	0.93 (0.16)	*+ 0.02*	*+ 0.01*
SAUS Cognition	0.95 (0.06)	0.98 (0.05)	0.95 (0.06)	0.97 (0.05)	0.95 (0.07)	*+ 0.03*	*0.00*
SAUS Pain	0.89 (0.12)	0.87 (0.25)	0.92 (0.07)	0.84 (0.25)	0.90 (0.09)	*-0.02*	*+ 0.01*
MAUS (Total score)	0.59 (0.24)	0.70 (0.20)	0.70 (0.11)	0.66 (0.25)	0.68 (0.17)	*+ 0.11*	*+ 0.09*

Table 2 shows that most values of the SAUS lie constantly between 0.90 and 1.00, which indicates that the functional health status of our sample was, in essence, good throughout the two-year observation period. There are two exceptions: the SAUS of *Hearing* and the SAUS of *Pain*. As for the latter, its slightly lowered values are likely explained by the fact that our sample included many elderly persons (> 60 years), in whom chronic pain may be frequent. The SAUS of *Hearing*, on the other hand, reflects the effect of VSB receipt on the functional status of hearing. It started from a low pre-operative level (0.59) and increased up to 0.70 at three months after processor activation. On subsequent time-points, only minor fluctuations occurred. Repeated measures ANOVA of the changes over time did not reveal significance: F = 2.64, df = 4, *p* = 0.10. As for clinical relevance of these changes (indicated by a difference of at least 0.03 between mean scores), all post-operative values of the SAUS *Hearing* indicate clinically relevant improvement compared to the pre-operative ones.

The HUI 3 multi-attribute utility score (MAUS) showed a slightly different course over time than the SAUS of *Hearing*. It started from the same level (0.59), raised to 0.70 at three months and at six months after processor activation, but then declined to 0.66 and 0.68 at the following time-points. Repeated measures ANOVA did not reveal significance for the change over time: F = 3.14, df = 1.74, *p* = 0.08. When evaluating the differences in terms of clinical relevance, we find that all pre-post differences of the MAUS are clinically relevant. In other words, at any time-point after processor activation, the generic HRQoL of VSB recipients has clinically relevantly improved compared to before implantation.

## Discussion

### Summary and interpretation of findings

Data about HRQoL of VSB patients are still sparse, and short-term data (< 6 months) and long-term data (beyond 12 months) are even missing. Against this background, our study aimed to assess HRQoL in a sample of patients with conductive or mixed hearing loss who received a VSB, over a two-year period. Two different types of HRQoL questionnaires were administered to the patients: a hearing-specific one (NCIQ) and a generic one (HUI 3).

Regarding the short-term data, we hypothesized that HRQoL improvement would be seen soon after implantation. Because the VSB transmits the sound acoustically to the inner ear, patients hardly have to get used to it and learn to cope with unfamiliar auditory impressions, but can soon enjoy the benefits of improved hearing. Findings from both questionnaires suggest that this hypothesis is correct. In our sample, an important improvement of the HRQoL was seen already after three months of device use. However, there were no (or only small) changes at later points in time. While our data do not show that HRQoL continues to improve beyond three months of device use, they do not rule it out either. However, it appears that further improvements of HRQoL, if they occur, are very slow.

While the pre-post differences of NCIQ scores of our sample were statistically significant, they were not significant with the HUI 3 scores (SAUS *Hearing* and MAUS). It is noteworthy that the other two studies that administered the HUI 3 to VSB patients, also reported that the pre-post changes of the MAUS failed to reach statistical significance. We have no clear idea how to explain why utility scores seem to defy statistical significance. At any rate, we must be cautious about dropping the null hypothesis of the statistical test (i.e. that there is no difference between pre- and post-implantation utility scores). Fortunately, we are in the position to interpret the changes of the HUI 3 scores by using another criterion: clinical relevance. Based on this criterion (according to which a mean difference of at least 0.03 is relevant), we can state that all post-implantation scores of the MAUS and the SAUS *Hearing* indicate a clinically relevant improvement compared to the pre-operative health status. Regarding the extent of the pre-post differences (between + 0.07 and + 0.13), we can even say that the improvement is *highly* clinically relevant.

Differences between post-implantation HUI 3 scores were largely irrelevant; again, this suggests that HRQoL may not change after three months of device use. However, there are two exceptions: between the *six month* and *12 month* points in time after processor activation, the SAUS *Hearing* improved relevantly (+ 0.04), while the same time the MAUS worsened relevantly (-0.04). This surprising finding can only be explained in the way that attributes other than Hearing (perhaps *Pain*) caused the worsening of the MAUS at time-point *12 months after processor activation*.

### Comparison to other studies

Comparability of our findings to those of other studies is limited as only few studies exist that investigated HRQoL in VSB patients and used the same measures as we did. For instance, no study so far assessed hearing-specific HRQoL in VSB patients using the NCIQ.

As for generic HRQoL assessed with HUI 3, two studies exist that reported the MAUS from VSB patient samples: Edfeldt et al. [12] and Lasaletta et al. [13]. Table 3 shows the MAUS scores reported in their articles and additionally the scores obtained in our VSB sample. The comparability of the scores is somewhat limited because the other two studies administered the HUI 3 to their sample only at one and two points in time after implantation, respectively.

**Table 3:** Results of pre-post comparisons of the Multi-attribute Utility score (MAUS) in three different VSB samples

MAUS	pre-operative	3 months	6 months	12 months	24 months	Maximum increase
Edfeld et al. [12]	0.57	-	0.65	-	-	+ 0.08
Lasaletta et al. [13]	0.60	-	0.69	0.75	-	+ 0.14
Present study	0.59	0.70	0.70	0.66	0.68	+ 0.11

Despite the missing scores in Table 3, some comparison is possible. Looking at points in time *pre-operative* and *six months after processor activation*, we see that the three samples present with quite similar values of the MAUS. They start at comparable levels of generic HRQoL (between 0.57 and 0.60) and increase to a similar extent (between + 0.08 and + 0.11) within six months. Beyond six months, however, the findings are inconsistent: while in the sample of Lasaletta et al. [13] the MAUS continued to increase, in our sample it declined a little. However, we must be cautious to interpret the observed changes, as in both studies the statistical tests failed to yield significance for the score differences. We are hence unable to comment on this inconsistency. It must be pointed out however, that the decline of the MAUS in our sample was due to deteriorations in attributes other than *Hearing*, namely Pain. As the MAUS is a measure for generic HRQoL, it reflects also changes in other health functions. Considering that our sample included many elderly patients, the long-term decline of the MAUS is likely due to age-related declines of various health functions of these patients.

## Conclusions

Implantation of a VSB is an effective treatment of hearing loss, resulting in a statistically significant increase of hearing-specific HRQoL and to a clinically relevant increase of generic HRQoL. The increase occurs quickly, i.e. within three months of device use. Beyond three months, only small fluctuations of HRQoL scores are seen, suggesting that the achieved level of HRQoL either remains stable or improves only slowly at subsequent points in time.
